# Dietary supplementation with Mexican foods, *Opuntia ficus indica, Theobroma cacao*, and *Acheta domesticus*: Improving obesogenic and microbiota features in obese mice

**DOI:** 10.3389/fnut.2022.987222

**Published:** 2022-12-02

**Authors:** Rebeca Rosas-Campos, Alejandra Meza-Rios, J. Samael Rodriguez-Sanabria, Ricardo De la Rosa-Bibiano, Karina Corona-Cervantes, Jaime García-Mena, Arturo Santos, Ana Sandoval-Rodriguez, Juan Armendariz-Borunda

**Affiliations:** ^1^Department of Molecular Biology and Genomics, Health Sciences University Center, Institute for Molecular Biology in Medicine and Gene Therapy, University of Guadalajara, Guadalajara, Mexico; ^2^Departamento de Genética y Biología Molecular, Cinvestav, Ciudad de México, Mexico; ^3^Tecnológico de Monterrey, EMCS, Guadalajara, Mexico

**Keywords:** *Opuntia ficus indica*, *Theobroma cacao*, *Acheta domesticus*, obesity, Mexican diet, obesogenic diet

## Abstract

**Introduction:**

An obesogenic diet, a diet high in saturated fats and sugars, is a risk factor for the development of multiple obesity-related diseases. In this study, our aim was to evaluate the effect of supplementation with a mixture of Mexican functional foods (MexMix), *Opuntia ficus indica* (nopal), *Theobroma cacao*, and *Acheta domesticus* (edible crickets), compared with a high-fat and fructose/sucrose diet on an obesogenic mice model.

**Methods:**

For this study, 18 male C57BL/6J mice were used, which were divided into three groups: (1) control group: normal diet (ND), (2) HF/FS group: high-fat diet along with 4.2% fructose/sucrose and water (*ad libitum* access), and (3) therapeutic group (MexMix): HF/FS diet up to week 8, followed by HF/FS diet supplemented with 10% nopal, 10% cocoa, and 10% cricket for 8 weeks.

**Results:**

MexMix mice showed significantly reduced body weight, liver weight, visceral fat, and epididymal fat compared with HF/FS mice. Levels of triglycerides, cholesterol, LDL cholesterol, insulin, glucose, GIP, leptin, PAI-1, and resistin were also significantly reduced. For identifying the gut microbiota in the model, 16S rRNA gene sequencing analysis was performed, and the results showed that MexMix supplementation increased the abundance of *Lachnospira, Eubacterium coprostanoligenes*, and *Blautia*, bacteria involved in multiple beneficial metabolic effects. It is noteworthy that the mice supplemented with MexMix showed improvements in cognitive parameters, as evaluated by the novel object recognition test.

**Conclusion:**

Hence, supplementation with MexMix food might represent a potential strategy for the treatment of obesity and other diseases associated with excessive intake of fats and sugars.

## Introduction

The World Health Organization defines obesity as excessive fat accumulation that could impair health due to an energy imbalance between calories consumed and calories expended (1). Obesity is associated with a decrease in life expectancy by 5–20 years (2). In the past 50 years, the prevalence of obesity has increased across the world, not only in adults but also among children and adolescents (3).

Nowadays, we are living in obesogenic environments that affect our lifestyle, including reduced home cooking, decreased physical activity, and overwhelming availability of processed snacks, soft drinks, and fast food (3). In consequence, the intake of fat and sugar has dramatically increased, especially among youngsters, which is one of the principal contributors to obesity (4), leading to a burden in health and the social ecosystem.

Criollo cacao bean is an ancient crop native to Mexico and Central and South America, along with maize. Cacao bean (*Theobroma cacao*) contains 12–18% of its dry weight of polyphenols and 60% of monomeric and oligomeric flavanols, making this fruit rich in flavanols (5). Cacao polyphenols have strong antioxidant property and a positive role in metabolic disorders, such as diabetes, and weight gain (6).

Nopal (*Opuntia ficus indica*) is a member of the *Cactaceae* family, native to the American continent, and a vegetable extensively consumed in Mexico (7). Nopal has been recognized as a functional food due to its high content of soluble and insoluble fibers, vitamin C (a well-known antioxidant), and phytochemicals like polyphenols, mainly flavonoids (7, 8). On the other hand, ethnic populations of the south of Mexico eat insects as part of their diet, including grasshoppers, crickets, and agave worms (9). Thus, Mexico is one of the countries that mostly consume and use insects as food in Latin America (10), and several companies produce and commercialize diverse foodstuffs prepared from insects (11).

Crickets (*Acheta domesticus*) are rich in protein, including all essential amino acids, fats, and minerals (12, 13). Furthermore, breeding of insects for human consumption has a lower environmental impact than traditional animal breeding since its production does not generate a large amount of carbon dioxide emissions. In addition, conventional livestock breeding requires a great deal of land, water, and food for their maintenance (14). Thus, the use of crickets as a source of proteins for humans represents an excellent choice.

The scientific literature reports that nopal and cocoa have moderate therapeutic effects on treating obesity (6, 7). Crickets have been proven as a sustainable source of protein; however, their impact on obesogenic features has not been described. Only one study involving healthy subjects has shown the protein source, tolerance, and edible characteristics of crickets, but it did not focus on obesity-related parameters (15). Evidence presented here is solely based on the individual use of the three components mentioned. Consequently, we hypothesized that a mixture of these three Mexican-origin foods would have additive or synergistic health benefits more than each individual nutriment. Therefore, the aim of this study was to evaluate the effect of supplementation of a mixture of *Opuntia ficus indica, Theobroma cacao*, and *Acheta domesticus* in lipid and obesogenic pathways in mice fed a high-fat/high-sugar diet.

## Materials and methods

### Animals and diet

For this study, 7-week-old male C57BL/6J mice weighing 20–25 g were fed a standard diet and water before the experiment. The mice were housed in a room with a 12-h/12-h light–dark cycle at a temperature of 22 ± 1°C. The animal protocols were in accordance with the Official Mexican Norm NOM-062-ZOO-1999, guidelines of the University of Guadalajara on animal care, and the criteria outlined in the Guide for the Care and Use of Laboratory Animals published by the NIH. The mice were given *ad libitum* access to beverage. After 1-week acclimatization, the mice were randomly allocated to three groups and fed either a standard diet (Envigo T.2018S.15) and water [NormoDiet group (ND), n = 6] or a high-fat diet (35% Kcal in fat) together with high-carbohydrate beverage (2.31% fructose, 1.89 % sucrose) (HF/FS groups) for 8 weeks. At the 8th week, HF/FS animals were divided into two subgroups (n = 6): the HF/FS control group was continued with the HF/FS diet until the 16th week to induce obesity, while six animals (MexMix group) received a supplementary diet with a mixture of foods of Mexican origin: *Opuntia ficus indica, Theobroma cacao*, and *Acheta domesticus* (10% w/w each) from the 8th week to 16th week. The animals were killed under anesthesia after 4 h of fasting. The epididymal adipose tissue was immediately collected and weighed. For histological examination, the epididymal fat pad was fixed with 4% paraformaldehyde. Compositions of the diets are summarized in Supplementary Table S1.

### Energy intake, weight, and insulin sensitivity

Food intake was measured systematically three times per week between 9:00 and 10:00 am, and energy intake was calculated from food and beverage consumed. Ingestion-related data were analyzed in two phases: before and during the dietary treatment. Weights of the mice were recorded weekly during the study. The mice fasted for 4 h before the insulin tolerance test (ITT), which was conducted 48 h before killing. Human-recombinant short-acting insulin was administered intraperitoneally at a dose of 0.75 U/kg. Serum glucose levels were measured at 0, 30, 60, and 90 minutes after insulin injection *via* the tail vein. The area under the curve (AUC) was calculated.

### Serum biomarkers and fat tissue histological analysis

Serum levels of triglycerides, total cholesterol, and LDL cholesterol were measured using automated equipment. Leptin, insulin, GIP, resistin, and PAI-1 were determined in serum using Bio-Plex Pro Mouse Diabetes 8-Plex Assay (BIO-RAD, USA), and adiponectin was determined using Bio-Plex Pro Mouse Diabetes Adiponectin Assay (BIO-RAD, USA). A pathologist blinded to the study performed a histological analysis in hematoxylin and eosin (H&E)-stained epididymal fat tissue.

The adipocyte size (μm^2^), cell hyperplasia, cell hypertrophy, and inflammatory infiltrates were analyzed. To assess the adipocyte size, at least 10 microscopic fields/mice at 20X magnification were analyzed using Image-Pro software (16).

### Microbiota diversity analysis

Fecal samples from the cecum were collected and stored at −80°C until analysis. Total DNA was extracted using the QIAamp Fast DNA Stool Mini Kit (QIAGEN, Hilden, Germany). DNA concentration was measured using a Qubit fluorometer, and purity was monitored in 1% agarose gels. DNA samples were sequenced using primers 349F (5 -CCT-ACG-GGN-GGC-WGC-AG-3 ) and 785R (5 -GGA-CTA-CHV-GGG-TAT-CTA-ATC-C 3 ) targeting the V3–V4 hypervariable regions of the 16S rRNA gene using an Illumina MiSeq system. Gut microbiome composition was assessed using QIIME2 (Quantitative Insights Into Microbial Ecology 2) software version 2021.8 (17). Sequences were denoised, filtered, and trimmed to 240 nt length using the DADA2 plugin using default settings following standard quality-filtering procedures (the removal of sequencing-related barcodes, sequences with a PHRED score < 20, and chimeric sequences) (18). All amplicon sequence variants (ASVs) were aligned by MAFFT (19), and a phylogeny was constructed using fasttree2 (20). Taxonomy was assigned to ASVs using the q2-feature-classifier (21) against the Silva_138 99% OTU reference sequences databases, and the relative abundance of ASVs was visualized in MicrobiomeAnalyst. An alpha rarefaction plot was visualized using QIIME2.

Beta diversity metrics were estimated using UniFrac analysis—weighted (22) and unweighted (23)—and permutational multivariate analysis of variance (PERMANOVA). A three-dimensional scatter plot was generated using principal coordinate analysis (PcoA) by QIIME2.

Alpha diversity was estimated using different indexes: observed number of species and Chao, Shannon, and Simpson indexes. These indexes were determined using the phyloseq package in R environment version 4.1.0 (24). The non-parametric Kruskal–Wallis test was used to examine alpha diversity metric dissimilarities. Also, a heat map of taxa abundance was constructed using the pheatmap package (version 1.0.12). Finally, Spearman correlation analysis was made using microbiome package (version 1.17.41) with bacterial taxa at level 6 and glucose-lipid data and presented as a heat map.

### Novel object recognition test

To evaluate the animal cognitive status, the novel object recognition (NOR) test was performed (25). In brief, the test consists of three steps: habituation, familiarization, and test. In the habituation phase, a day before the test phase, the mice were placed in a plastic box (32 x 52 x 30 cm), which was different from their habitual cage, for 30 min. On the next day, in the familiarization phase, each mouse was placed in the plastic box for 10 min with two identical objects; 90 minutes later, each mouse returned to the plastic box with one “familiar” object and a new object (different in shape and color). The time spent with each object was measured. “Exploration” was defined when the nose of the mice was 2 cm or less away from the object. The preference index and the discrimination index were calculated using the following formulas:


$$\begin{array}{l} \text{Preference index }\left(\%\right)\\ \;=\text{Time new object}/\left(\text{Time new object }+\text{ Time familiar object}\right)\\ \text{Discrimination index }\left(\%\right)\\ \;=\left(\text{Time new object }-\text{ Time familiar object}\right)\\ \;/\left(\text{Time new object }+\text{ Time familiar object}\right) \end{array}$$

### Statistical analysis

Data are presented as mean ± SD or mean ± SEM for graphical representations. The Shapiro–Wilk test was used to establish the normality of variables. For variables with a normal distribution, statistical significance was determined by parametric one-way ANOVA and Tukey's *post hoc* test. For variables with a non-normal distribution were determined by using the non-parametric Kruskal–Wallis test. Data were analyzed using SPSS 20 software and GraphPad Prism 5.0. A *P*-value of < 0.05 was considered statistically significant.

## Results

### MexMix diet decreases calorie intake

Calorie intake from food and beverage were used to calculate the total daily energy intake. Table 1 indicates that daily energy intake was higher in HF/FS and MexMix groups than in the ND group (p = 0.014). It is noteworthy that there were no differences in energy intake between HF/FS and MexMix during the period of obesity induction. During the treatment, MexMix animals reduced their food and calorie intake compared with the HF/FS group (*p* < 0.001). ND and HF/FS groups displayed a similar behavior of food or energy intake after and during the treatment. MexMix mice reduced the quantity of food and calorie intake during the treatment period compared with that prior to the treatment (*p* < 0.001).

**Table 1:** Dietary intake during experimental phases.

		**ND**	**HF/FS**	**MexMix**
**Prior treatment**				
Daily energy intake	Kcal	13.12 ± 1.25^a, ns^	16.24 ± 3.03^b, ns^	15.90 ± 1.48^b^,**
Daily food intake	g	4.23 ± 0.41^ns, ns^	3.91 ± 0.72^ns, ns^	3.78 ± 0.32^ns^,***
Diet fat percentage	%	6.2	34.13	34.13
Daily fat intake	Kcal	2.42 ± 0.24^a, ns^	5.44 ± 1.07^b, ns^	5.08 ± 0.43^b^,**
**During treatment**				
Daily energy intake	Kcal	12.06 ± 1.47^a, ns^	15.80 ± 1.39^b, ns^	13.26 ± 1.61^a^,**
Daily food intake	g	3.89 ± 0.47^a, ns^	3.73 ± 0.36^a, ns^	3.17 ± 0.34^b^,***
Diet fat percentage	%	6.2	34.13	33.18
Daily fat intake	Kcal	2.17 ± 0.26^a, ns^	5.00 ± 0.49^b, ns^	3.77 ± 0.41^c^,**

Values represent mean ± SD.

ns: no significant differences.

a, b, c: Differences between groups, ANOVA *post hoc* Tukey.

**, ***: Prior treatment versus during treatment, *T*-student paired.

### Supplementation reduces fat accumulation and liver weight and improves insulin resistance

**Figure 1:**
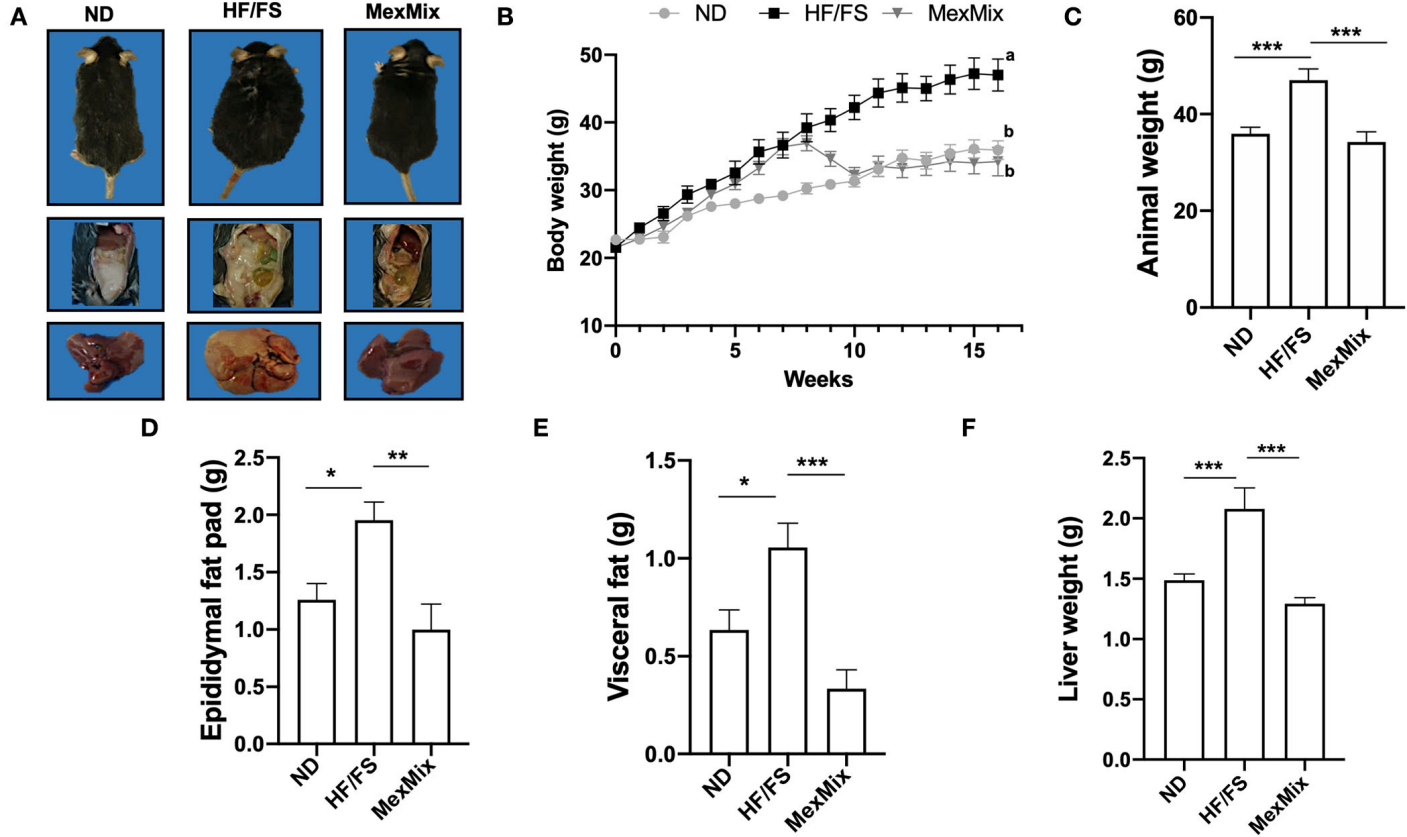
Effect of MexMix on the body and liver weight and fat pads. **(A)** Representative images of the mice, epididymal fat, and liver from each group. **(B)** Body weight gain of 16 weeks. **(C)** Body weight while killing. **(D)** Epididymal fat pad weight. **(E)** Visceral fat weight. **(F)** Liver weight. Data represent mean ± SEM (*p < 0.05; **p < 0.01; *** *p* < 0.001). a, b: Differences between groups.

As shown in Figure 1A, HF/FS animals developed obesity and displayed abundant intra-abdominal adipose tissue and a pale and steatotic liver. MexMix animals had a body mass and intra-abdominal fat pads similar to ND mice, as well as normal liver appearance. The HF/FS-diet group showed significant weight gain compared with ND and MexMix groups (Figure 1B). The mice fed the MexMix diet showed a significant reduction in body weight as soon as 2 weeks after the beginning of supplementation, reaching weights similar to ND animals. While killing, the body weight of the mice in the MexMix group was significantly reduced compared with that of the HF/FS mice (33.63 ± 6.34 vs. 43.67 ± 5.57g; *p* < 0.001) (Figure 1C). As depicted in Figures 1D,E, the epididymal fat pad (1.19 ± 0.70 g vs. 1.95 ± 0.35; *p* = 0.003) and visceral fat (0.30 ± 0.23 g and 1.06 ± 0.28; *p* < 0.001) were statistically reduced in the MexMix group compared with those in the HF/FS mice. For determining steatosis, liver weight was measured, and statistical differences were found between HF/FS (2.08 ± 0.39 g) and MexMix (1.29 ± 0.13 g; *p* < 0.001) groups (Figure 1F).

**Figure 2:**
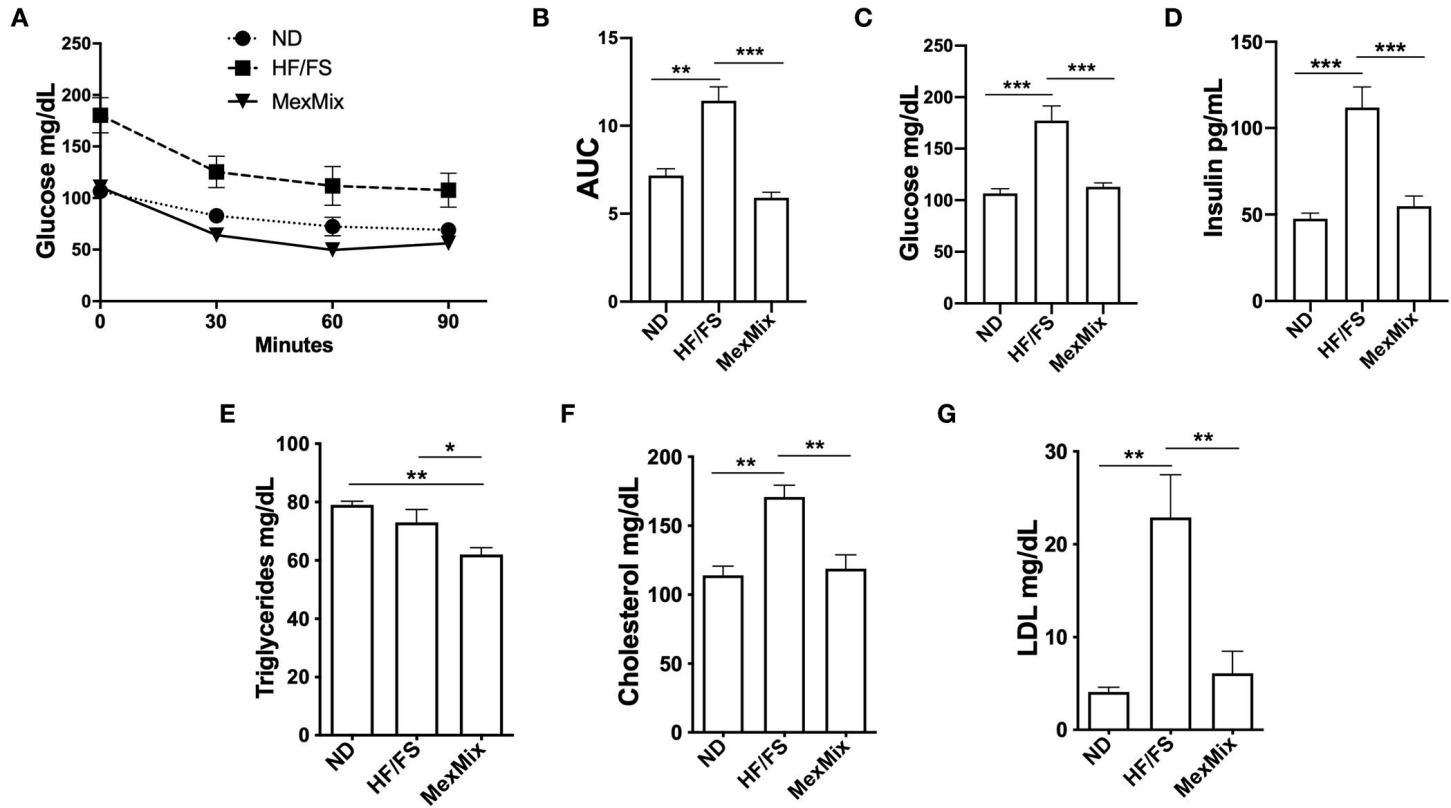
Effect of MexMix diet in glucose and lipid metabolism. **(A)** ITT test: glucose serum levels at 0, 30, 60, and 90 minutes. **(B)** Total area under the curve calculated from the ITT data. **(C)** Serum glucose levels. **(D)** Serum insulin levels. **(E)** Triglyceride levels. **(F)** Cholesterol levels. **(G)** LDL cholesterol levels. Data are expressed as mean ± SEM (*p < 0.05; **p < 0.01; *** *p* < 0.001).

The MexMix mice displayed a significant increase in insulin sensitivity in the ITT (Figures 2A,B), as shown by a decrease in the area under the curve value (AUC) (*p* < 0.001). The lowest AUC values were observed in the animals fed a MexMix diet (5.91 ± 0.69) compared with those in the HF/FS mice (11.44 ± 1.74; *p* < 0.001). The treated group showed no difference in the ND mice (7.29 ± 0.94 AUC) (Figure 2B).

As shown in Figure 2C, glucose serum levels at the 16th week were reduced (*p* < 0.001) in ND (106.60 ± 10.06 mg/dL) and MexMix (113.0 ± 9.88 mg/dL) groups compared with those in the HF/FS group (145.80 ± 8.98 mg/dL). As shown in Figure 2D, insulin levels in ND animals before treatment were similar to those after MexMix treatment (47.50 ± 8.72 and 54.83 ± 17.63 pg/mL, respectively), while HF/FS mice remained hyperinsulinemic (111.9 ± 29.13 pg/mL; *p* < 0.001).

### MexMix diet improves serum lipid markers and adipokines

**Figure 3:**
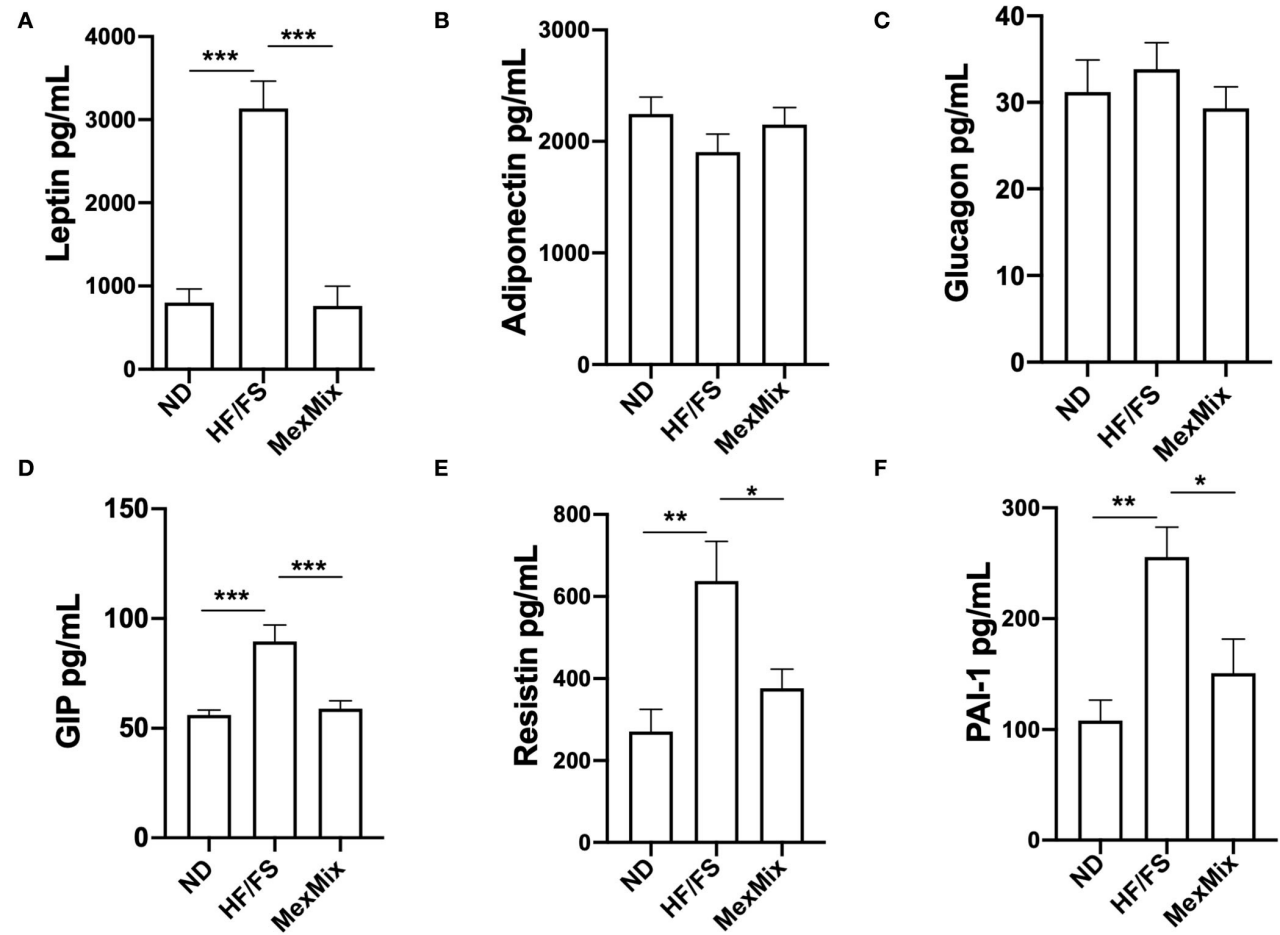
Effect of MexMix diet in serum adipokines. **(A)** Leptin levels. **(B)** Adiponectin levels. **(C)** Glucagon levels. **(D)** GIP values. **(E)** Resistin levels. **(F)** PAI-1 values. Data represent mean ± SEM (*p < 0.05; **p < 0.01; *** *p* < 0.001).

After the intervention, serum levels of TG, cholesterol, and LDL cholesterol showed changes in the MexMix group, indicating metabolic improvement. As shown in Figure 2E, TG values in MexMix mice showed a significant reduction (62.0 ± 6.3 mg/mL) compared with those in the HF/FS and ND animals (73.0 ± 8.98 mg/mL; *p* = 0.046 and 79.0 ± 2.58 mg/mL; *p* = 0.003, respectively). Figure 2F displays that cholesterol levels in the ND and MexMix animals showed similar levels (114.0 ± 15.0 and 118.9 ± 26.44 mg/mL) but were reduced in the HF/FS group (170.8 ± 19.1 mg/mL, *p* = 0.003). Also, LDL cholesterol levels were significantly reduced after MexMix therapy (6.11 ± 5.85 mg/mL; *p* < 0.001), while the HF/FS animals displayed a cholesterol level of 22.9 ± 10.25 mg/mL (Figure 2G). Serum adipokines were also quantified, showing that leptin, as expected, is highly increased (3,134.0 ± 809.5 pg/mL; *p* < 0.001) in the HF/FS animals (Figure 3A), while leptin levels in the MexMix mice were comparable with those in the ND mice (801.6 ± 431.8 and 760.2 ± 712.9 pg/mL). Adiponectin and glucagon serum levels did not show statistical differences between the groups (Figures 3B,C), although adiponectin showed a tendency to increase in the treated mice. GIP and resistin revealed a significant reduction in the MexMix mice (58.88 ± 10.28 pg/mL; *p* < 0.001 and 376.6 ± 138.7 pg/mL; *p* = 0.024) compared with those in the HF/FS group (89.50 ± 15.07 and 637.4 ± 236.9 pg/mL) (Figures 3D,E). As depicted in Figure 3F, PAI-1 levels in the MexMix-fed animals showed a significant decrease compared with those in the HF/FS group (150.9 ± 81.21 vs. 255.7 ± 59.96 pg/mL; *p* = 0.041), with values close to ND.

### MexMix diet reduces body adiposity and adipose tissue inflammation

**Figure 4:**
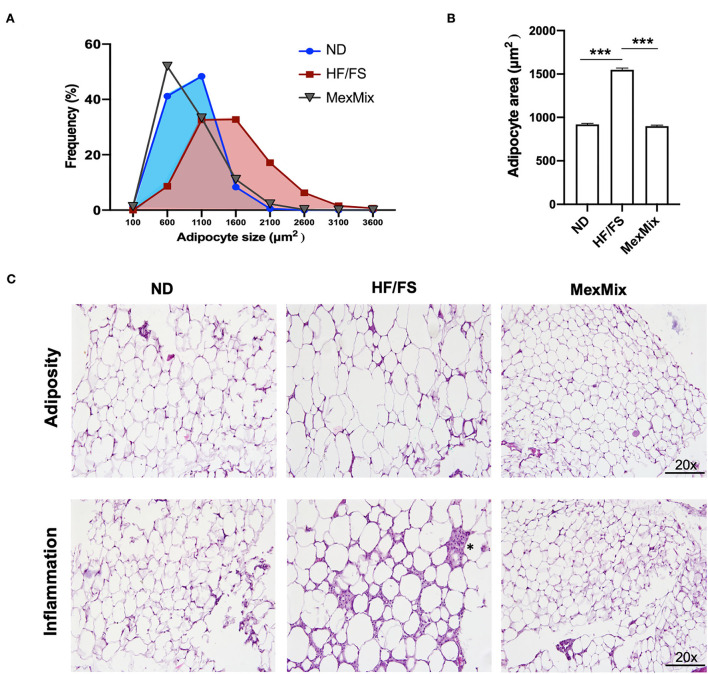
Effect of the MexMix diet on adipocyte size. **(A)** Frequency of adipocyte size. **(B)** Mean adipocyte area. **(C)** Hematoxylin and eosin staining [adipocyte area and inflammatory infiltrate (*)]. Data represent mean ± SEM (*** *p* < 0.001).

Approximately 58% of cells had a surface area >1600 μm^2^ in the HF/FS group, whereas in the MexMix group, only 13% of cells had a surface area >1,600 μm^2^ (Figure 4A). The adipocyte size significantly decreased 1.6-fold in the MexMix animals (926.91 ± 266.75 μm^2^) compared with that in the HF/FS mice (1548 ± 581.1μm^2^; *p* < 0.001). No statistical difference was found between the ND (919.2 ± 299.2 μm^2^) and MexMix (899.2 ± 377.8 μm^2^) groups (Figure 4B). As shown in Figure 4C, the ND group presents mature adipocytes of small and uniform size and form; the cytoplasmatic membranes are well defined with a large and compressed cytoplasm, presenting a single fat vacuole. No inflammatory infiltrate was observed. In the HF/FS animals, mature adipocytes with great variations in size and shape were observed. Most cells were two times as large as others, suggesting a process of hyperplasia. Areas with an increased extracellular matrix were observed, secondary to a chronic inflammatory infiltrate of mononuclear predominance (^*^) and congestive blood vessels. The MexMix diet prevented and reverted the effect of high fat and high fructose/sucrose. The 8-week treatment restored the form and size of adipocytes, also histological characteristics are similar to ND mice. Compared with the HF/FS group, the MexMix animals showed significantly reduced inflammatory infiltrates.

### *Lachnospira, Eubacterium coprostanoligenes*, and *Blautia* are enriched after MexMix diet treatment

**Figure 5:**
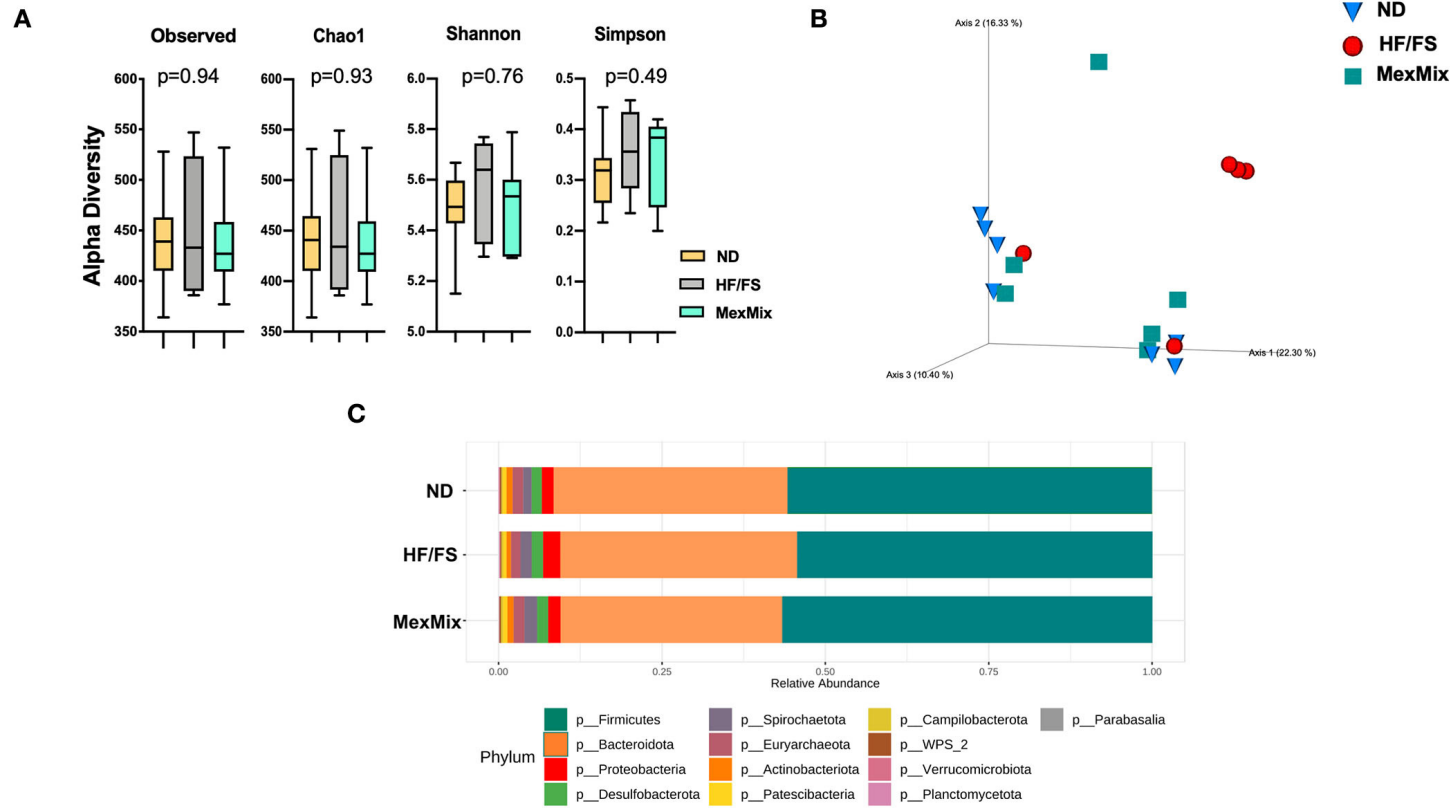
Effect of MexMix in microbiota diversity. **(A)** Alpha diversity: observed ASVs, Chao1, Shannon, and Simpson indexes. **(B)** Beta diversity and three-dimensional scatter plot generated using principal coordinate analysis (PCoA). **(C)** Relative abundance of bacterial phyla. Data represent mean ± SEM.

**Figure 6:**
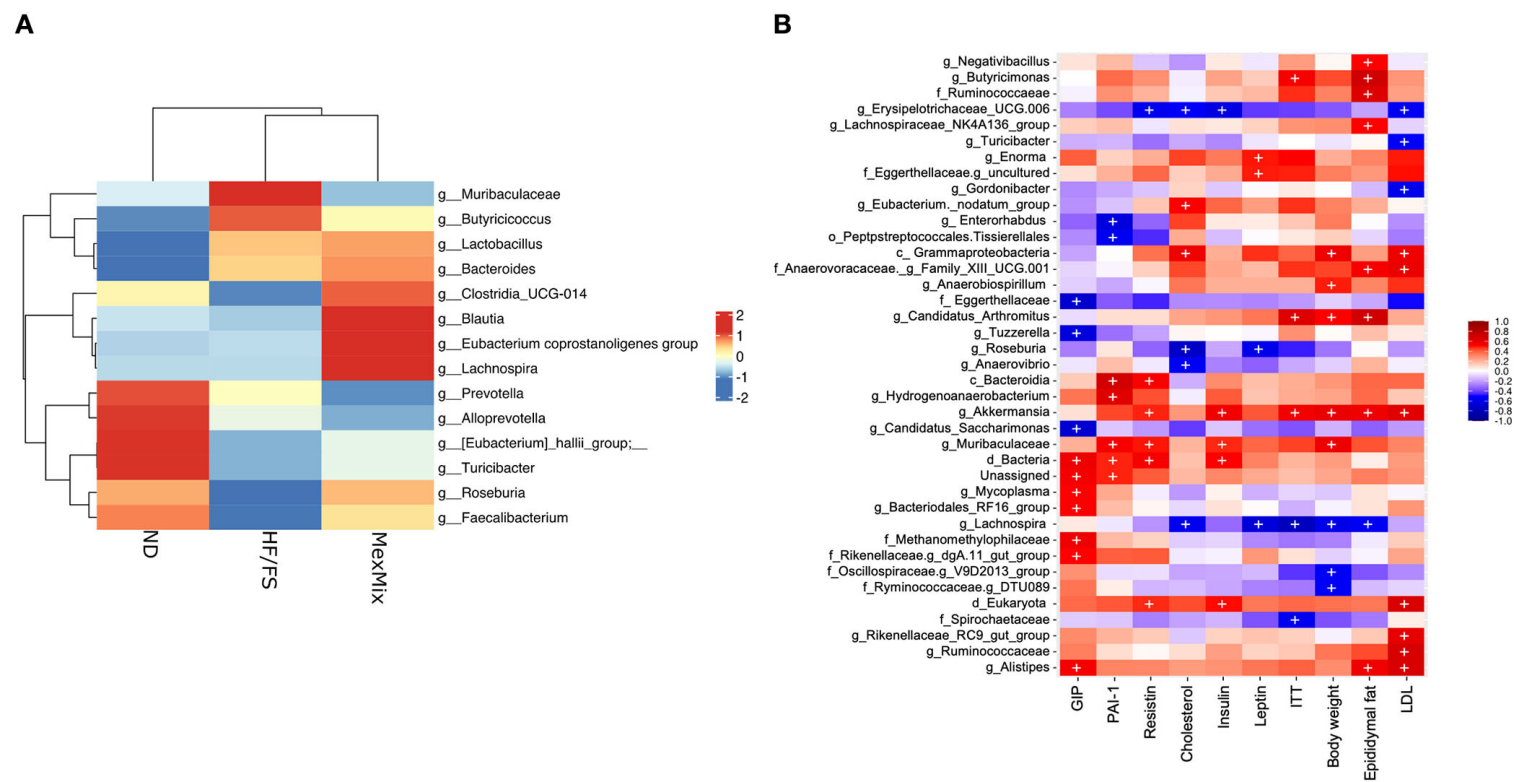
**(A)** Heat map with relative abundance with enriched genus. **(B)** Heat map with Pearson's correlation between several taxa and parameters related to glucose and lipid metabolism. Statistically significant (*p* < 0.05) correlations are represented by (+).

We characterized the bacterial diversity in stool samples collected during euthanasia using Illumina NGS of regions V3–V4 of the 16S rRNA. In brief, we obtained 2,755,409 raw sequence reads from 18 analyzed samples, and 1,135,792 feature counts after correction with DADA2 (Supplementary Table S2). The alpha rarefaction curves indicated that the depth of sequencing among the samples was sufficient to process the data (Supplementary Figure S1). Alpha diversity was estimated by observed ASVs, Chao1, Shannon, and Simpson indexes, which showed similar results and no differences between the ND, HF/FS, and MexMix groups (*p* = 0.94, *p* = 0.93, *p* = 0.76, *p* = 0.49, respectively, for the Kruskal–Wallis test) (Figure 5A). Regarding beta diversity, the microbial communities of ND controls and MexMix did not cluster separately, according to UniFrac metrics in a PCoA plot, while the HF/FS animals showed a tendency to cluster separately from ND and MexMix samples (Figure 5B). The PERMANOVA did not show a statistically significant difference between the groups (MexMix vs. ND p= 0.85; MexMix vs. HF/FS p= 0.62). The relative abundance of the three main phyla, namely, Firmicutes, Bacteroidetes, and Proteobacteria, represented approximately 94% of the sequences at the phylum level. The relative abundance of the phylum Firmicutes was at least 59%, and that of Bacteroidetes was at least 29% in all groups. The phylum Proteobacteria showed an increase in the HF/FS group and tends to decrease in the ND and MexMix groups. However, none of the phylum among the groups showed significant differences (Figure 5C, Supplementary Table S3). The analysis at the family level showed that *Prevotellaceae, Lachnospiraceae*, and *Lactobacillaceae* families were more abundantly present in all groups (Supplementary Figure S2). At the genus level, supplementation with MexMix increased the abundance of *Lachnospira, Eubacterium coprostanoligenes*, and *Blautia*, which are associated with multiple beneficial metabolic effects, whereas the level of *Muribaculaceae, Prevotella*, and *Alloprevotella* tended to decrease (Figure 6A).

On the other hand, we found an important correlation between fecal microbiota and several parameters related to glucose and lipid metabolism (Figure 6B). The genus *Lachnospira* showed negative correlations with weight, epididymal fat, serum leptin, cholesterol, and AUC-ITT. *Muribaculaceae* and *Akkermansia* genera showed a positive correlation with increased resistin, insulin resistance, epidydimal fat, LDL cholesterol, and body weight; besides, the class *Gammaproteobacteria* showed a positive correlation with increased body weight and levels of cholesterol and LDL cholesterol (Figure 6B).

### Supplementation with *Opuntia ficus indica, Theobroma cacao*, and cricket prevented a decline in novel object recognition in mice fed a high-fat diet

**Figure 7:**
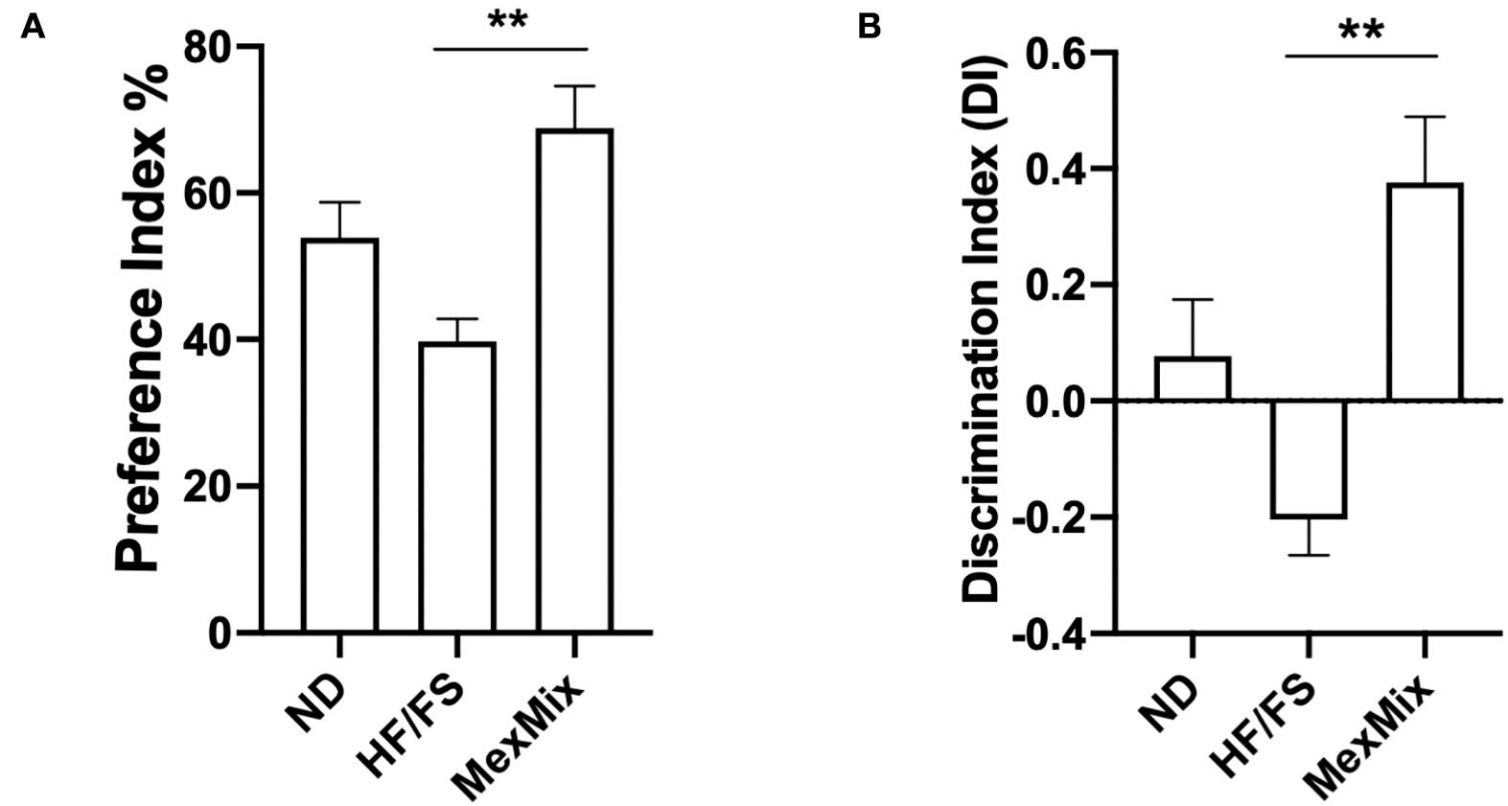
Effect of MexMix diet on the novel object recognition (NOR) test. **(A)** Preference index. **(B)** Discrimination index. Data are expressed as mean ± SEM (**p < 0.01).

During the NOR test, the MexMix mice spent more time exploring the new object, after the familiarization step, with a preference index (PE) of 68.8 ± 12.82%, a value significantly different from the that in the HF/FS group (39.72 ± 6.84%; *p* = 0.004) (Figure 7A). This result showed that the MexMix diet could prevent HF/FS diet-induced long-term memory deficit. The HF/FS mice spent less time exploring the novel object, indicating memory impairment shown by a negative discrimination index (DI) (Figure 7B).

## Discussion

Obesity was absent in the Mesoamerican population, whose diet was based on corn, black beans, nopal, chili, pumpkin, chia seeds, turkey, fish, fowl, and insects (26). Currently, obesity is a prevalent disease in most occidental populations as lifestyle has dramatically changed. Reduction in the calorie intake is one of the main therapeutic approaches for obesity, but also is an indication with low therapy adherence. Here, we tested if a high-calorie diet supplemented with 10% nopal, cacao, and crickets shows beneficial effects in a mouse model.

In our study, the MexMix diet counteracted the negative outcomes of the obesogenic diet, reducing fat accumulation in fat pads and the liver, restoring insulin sensitivity, and improving metabolic and cognitive parameters. Remarkably, we found that the MexMix mice reached a comparable body weight to the ND mice in 2 weeks (Figure 1B), but was not achieved in rats fed a high-fat-sucrose diet and supplemented only with 5% nopal fiber (27). On the other hand, in an obesity mice model of 4 weeks, a similar body weight was found between the ND group and mice fed a high-fat cocoa-enriched diet (10%) (28); a study by Gu et al. for a longer period (18 weeks; similar to our model) found that cacao treatment (8%) in HF/FS-fed mice showed higher weight than the ND group (29). In addition, we reported analogous glucose and insulin levels between the ND and MexMix mice (Figures 2C,D), as opposed to previous studies showing significantly decreased glucose and insulin levels by individual supplementation of nopal and cacao compared with the HF/FS group; however, the levels in the treatment group were consistently higher than those in ND groups (27, 30–32). Then, our results obtained with the mixture of nopal, cacao, and cricket in this study suggest an additive effect.

Since the mice fed the MexMix diet showed a slightly decreased food intake (Table 1), we believe this can be explained in part by the elevated quantity of fiber (~9%) present in the MexMix diet. It has been shown that there is an association between dietary fiber intake and satiety in animal models and human studies (33); supplementation with dietary fibers has reported a reduction in daily food intake (34–37). The MexMix group showed a decrease in the total Kcal and fat Kcal consumed per day, suggesting that the therapeutic calorie restriction indicated in the management of patients with obesity as a nutritional intervention can be achieved using this supplementation (38, 39).

On the other hand, the MexMix diet decreased serum lipid levels, including total cholesterol, LDL cholesterol, and TGs (Figures 2E–G). A previous study showed that rats fed an HF/FS diet supplemented with 5% w/w nopal for 1 month showed a substantial decrease in the levels of cholesterol, LDL cholesterol, and TGs (27). Also, female Sprague–Dawley rats fed a high-fat methionine–choline deficient diet supplemented with 12.5% cocoa powder for 28 days showed reduced levels of circulating triglycerides, and extended supplementation for 56 days resulted in even lower TG levels (40). In addition, nopal and cacao have independently improved insulin sensitivity in experimental and clinical scenarios. *Theobroma cacao* administration for 10 weeks significantly reduced animal weight, glycemia, insulinemia, and insulin resistance in a rat model of DM2 (32). In healthy individuals and patients with DM2, intake of 50 g of dehydrated nopal or 300 g of steamed nopal, respectively, reduced levels of postprandial blood glucose, serum insulin, GIP, and increased antioxidant activity (41). Our mix, then, exhibited an additive beneficial effect of both compounds, nopal and cacao.

The fat cell size positively correlated with impaired whole-body metabolic regulation and systemic insulin resistance (42). Here, we reported a significant reduction in the adipocyte diameter in the MexMix group (Figure 4B). An earlier study showed that supplementation with 4% nopal-derived dietary fiber showed reduced adipocyte size in HF/FS-fed rats compared with HF/FS controls (30). In the C57BL/6J mice, HF/FS diet supplementation with unsweetened cocoa powder (8%) for 18 weeks did not affect the epididymal fat pad or total visceral adiposity; however, cocoa treatment did influence adipocyte cell size distribution and reduced fat tissue inflammation (29). In this context, Stull et al. demonstrated a reduction of TNF-α plasma levels using cricket powder in the diet, suggesting reduced systemic inflammation (15). Bearing that in mind, the nutrient mix developed in our study preserved and enhanced the properties that reduced histological fat tissue inflammation.

Dysfunction of adipose tissue is characterized by the upregulation of pro-inflammatory adipokines (43). The MexMix treatment decreased serum levels of leptin, GIP, and resistin (Figures 3A–E, respectively). Accordingly, Moran-Ramos et al. showed that the concentration of leptin was significantly 38% decreased in rats fed an HF/FS diet with 4% nopal (30). In the present study, with the simultaneous supplementation of nopal, cacao, and cricket, we showed a 78% reduction of pro-inflammatory adipokines. Then, simultaneous supplementation showed better effects than individual nutraceuticals. In addition, supplementation with 4% nopal-derived dietary fiber did not change the levels of adiponectin, an anti-inflammatory adipokine (31). Our results showed that adiponectin tend to increase. Also, we found a reduction in PAI-1 levels by MexMix supplementation (Figure 3F). A previous study showed that higher PAI-1 levels were associated with systemic insulin resistance in individuals with obesity (44).

Metabolic dysfunction, caused by obesity, is also reflected in gut microbiota composition. However, some authors have shown inconsistencies in bacterial populations (45). Here, we did not find that MexMix influences alpha and beta diversities or phylum relative abundances. Nopal or cacao supplementation had been shown to change the microbiota composition in the HF/FS diet-fed rat and Zucker rat DM2 models, respectively (27, 32). Since fat intake in mice is recommended to be around 18% (46, 47), the diet with 34.13% fat (2X of the nutritional recommendation) we used was enough to cause obesity and metabolic alterations, but not dysbiosis; that is why we neither observed significant changes in alpha and beta diversities in HF/FS animals and nor between groups. However, we did notice an increase in the abundance of the genera *Eubacterium coprostanoligenes, Lachnospira*, and *Blautia* in the MexMix animals (Figure 6A), bacteria associated with multiple beneficial metabolic effects. Li et al. reported that feeding with *Eubacterium coprostanoligenes* decreases blood cholesterol concentration in germ-free mice (48), and several members from the genus *Lachnospira* are associated with SCFA production, like acetate and lactate (49, 50). The abundance of the genus *Blautia* showed a significant negative relationship with visceral fat accumulation in Japanese people (51). Enrichment of this genus is beneficial and, as shown in our results, could be associated with an improvement in metabolic parameters. Besides, this enrichment has been associated to fiber or flavonoids intake; abundant nutrients in nopal and cacao. For instance, supplementation with oroxylin A, a natural flavonoid, increased the levels of *Eubacterium coprostanoligenes* (52). Also, the abundance of *Lachnospira* increased in patients with overweight/obesity who were fed fiber supplementations for 12 weeks (53) or 10 weeks (54) and showed a negative correlation with patients' body weight (53). Like our data, Alvarez-Silleros et al. found that supplementation with a 10% cocoa-rich diet for 10 weeks in Zucker diabetic fatty rats increased the relative abundance of acetate-producing genus *Blautia*; in addition, they found a reduction in the abundance of Proteobacteria by cocoa treatment (32)—a tendency also present in our samples. In addition, the supplementation with 494 mg of cocoa flavanols per day for 4 weeks increased bifidobacterial and lactobacilli populations in healthy humans (55). Particularly, Sánchez-Tapia et al. fed rats with a high-fat diet (45%) along with 5% of dietary fiber from nopal and found an increase in the alpha diversity and enrichment of bacteria involved in SCFA production (27). Moreover, 1-month supplementation with boiled nopal 300 g/day in patients with obesity showed an increasing tendency in *Prevotella* enrichment, while *Roseburia* and *Eubacterium* enrichment were detected in patients with normal weight (56). Also, cricket powder supplementation (25 grams/day) for 14 days in healthy adults did not modify the alpha and beta diversities, but supported the growth of the probiotic bacterium *Bifidobacterium animalis* (15). On the other hand, *Lachnospira*, a well-known SCFA producer, negatively correlated with serum leptin, cholesterol, and ITT levels (Figure 6B). It has been described that SCFA dietary supplementation induced the activation of adipose and hepatic PPARγ, which modulates lipid metabolism through increased energy expenditure (57). While treating diabetic rat with acetate reduces weight gain and improves glucose tolerance (58).

Obesity has been associated with an increased occurrence of central disorders such as depression and impaired cognitive function (59). The mice fed an HFD (40% kcal) for 21 weeks clearly displayed impaired recognition memory by the NOR test (60). The ND and HF/FS groups showed a similar NOR index (Figures 7A,B) likely because our diet contains less fat (34%). This lack of difference was also reported in the C57BL/6 mice fed an HFD (60% kcal) for 5 weeks (61). However, the MexMix animals showed a statistically significant improvement in the memory recognition test. Several studies confirmed that acute and chronic intake of cocoa have a positive effect on several cognitive outcomes (62). Rats fed a diet supplemented with 0.05% theobromine, a primary methylxanthine found in cacao beans, resulted in a higher novel object preference, with a PE > 50% (63). Here, we report a PE of >65% with simultaneous supplementation of cacao, nopal, and cricket. In addition, supplementation with 5% nopal for a month decreased brain oxidative stress and neuroinflammation and improved cognitive function but decreased malondialdehyde (MDA) concentration, a marker of oxidative stress, and reduced neuroinflammation (27). Again, our data showed a higher percentage in cognitive tests, probably by the combined action of the three components.

In conclusion, diet supplementation with a mix of *Opuntia ficus indica, Theobroma cacao*, and *Acheta domesticus* improved obesity parameters caused by a diet rich in fat and simple carbohydrates. This dietary indication could be a non-pharmaceutical approach for the treatment of Western diet-induced obesity and associated comorbidities.

## Data availability statement

The data presented in the study are deposited in the NCBI BioProject repository, accession number PRJNA870154.

## Ethics statement

The animal study was reviewed and approved by Ethics, Research and Biosafety Committee, Health Sciences University Center, University of Guadalajara with number 20-122.

## Author contributions

RR-C contributed to the murine model, performed experiments, analyzed the data, and wrote the manuscript. AM-R involved in conceptualization, analyzed the data, and wrote the manuscript. JR-S contributed to the murine model. RR-B helped with the murine model and performed histological analyses. KC-C and JG-M contributed to microbiota analysis and reviewed and edited the manuscript. AS reviewed and edited the manuscript. AS-R helped with conceptualization, performed experiments, supervised the study, and reviewed and edited the manuscript. JA-B contributed to the conceptualization, funding acquisition, supervision, review, and editing. All authors contributed to the article and approved the submitted version.

## Funding

The (COETCyJAL) funded this project through FODECyJAL through the Grant No. 7941-2019 to JA-B.

## Conflict of interest

The authors declare that the research was conducted in the absence of any commercial or financial relationships that could be construed as a potential conflict of interest.

## Publisher's note

All claims expressed in this article are solely those of the authors and do not necessarily represent those of their affiliated organizations, or those of the publisher, the editors and the reviewers. Any product that may be evaluated in this article, or claim that may be made by its manufacturer, is not guaranteed or endorsed by the publisher.
