# Supplementary material for: Dietary supplementation with Mexican foods, Opuntia ficus indica, Theobroma cacao, and Acheta domesticus: Improving obesogenic and microbiota features in obese mice
Source: Front Nutr. 2022 Dec 2;9:987222. doi: 10.3389/fnut.2022.987222 (PMC9755723; doi:10.3389/fnut.2022.987222)
Supplement: Supplementary file 1 [file Data_Sheet_1.docx]

Supplementary Material

# Supplementary tables

|  | **ND** | | **HF/FS** | | **MexMix** | |
| --- | --- | --- | --- | --- | --- | --- |
|  | **g/100gr** | **% Kcal** | **g/100gr** | **% Kcal** | **g/100gr** | **% Kcal** |
| **Proteins** | 18.6 | 24 | 21.05 | 21.40 | 19.15 | 19.47 |
| **Carbohydrates** | 44.2 | 58 | 35.75 | 36.34 | 46.68 | 47.46 |
| **Fat** | 6.2 | 18 | 14.92 | 34.13 | 14.46 | 33.08 |
| **Nopal** | - | - | - | - | 10 | - |
| **Cricket** | - | - | - | - | 10 | - |
| **Cacao** | - | - | - | - | 10 | - |
| **Total fiber** | 3.5 | - | 4.26 | - | 9.19 | - |
| **Kcal/gr** | 3.1 | | 3.93 | | 3.61 | |
| **Water (2.31% fructose, 1.89 % sucrose)** | No | | Yes | | Yes | |

**Table S1:** Diets and drink water composition

**Table S2:** Sequences summary (n=18)

| **Parameter** | **Fecal samples n=18** |
| --- | --- |
| Total of raw read sequences | 2,755,409 |
| mean | 153,078.28 |
| min-max | 124,478 – 192,346 |
|  |  |
| Number of features ^1^ | 2,813 |
| Feature counts | 1,135,792 |
| count mean | 63,099.56 |
| count min–max | 52,719-77,888 |

^1^Summary of sequences after trimming at 240 nt and denoized with dada2.

**Table S3:** Relative abundance of phyla among groups

| **Phylum** | **ND** | **HF/FS** | **MexMix** | **p-value** |  |
| --- | --- | --- | --- | --- | --- |
| Firmicutes | 60.67 ± 7.07 | 59.25 ± 6.34 | 61.23 ± 8.11 | 0.96 |  |
| Bacteroidetes | 31.51 ± 6.54 | 32.19 ±5.82 | 29.82 ± 7. 46 | 0.71 |  |
| Proteobacteria | 1.72 ± 0.69 | 2.50 ± 1.18 | 1.86 ± 0.96 | 0.36 |  |
| Eutyarchaeota | 1.35 ± 0.22 | 1.25 ± 0.56 | 1.35 ± 0.43 | 0.76 |  |
| Actinobacteriota | 1.02 ± 0.34 | 0.76 ± 0.48 | 0.99 ± 0.50 | 0.43 |  |
| Desulfobacterota | 1.41 ± 0.62 | 1.51 ± 0.53 | 1.48 ± 1.04 | 0.86 |  |
| Spirochaetota | 1.05 ± 0.45 | 1.29 ± 0.75 | 1.62 ± 0.37 | 0.11 |  |
| Others | 1.36 ± 0.59 | 1.27 ± 0.54 | 1.65 ± 0.95 | 0.79 |  |

Data are represented as mean ± standard deviation. p-values were calculated using Kruskal Wallis test.

# Supplementary Figures

#
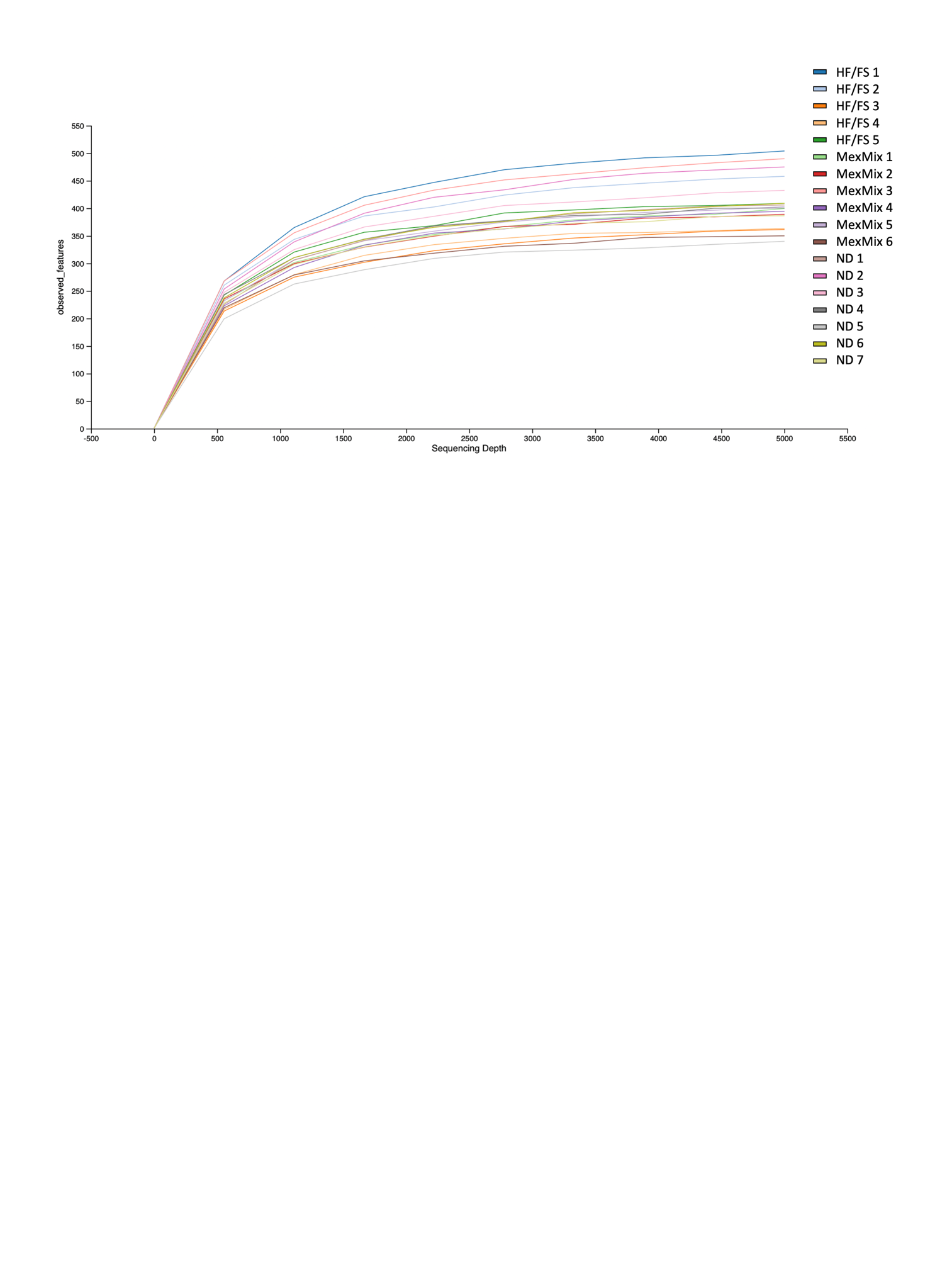


**Supplementary Figure 1.** Rarefaction plot of high-throughput DNA sequencing.

**
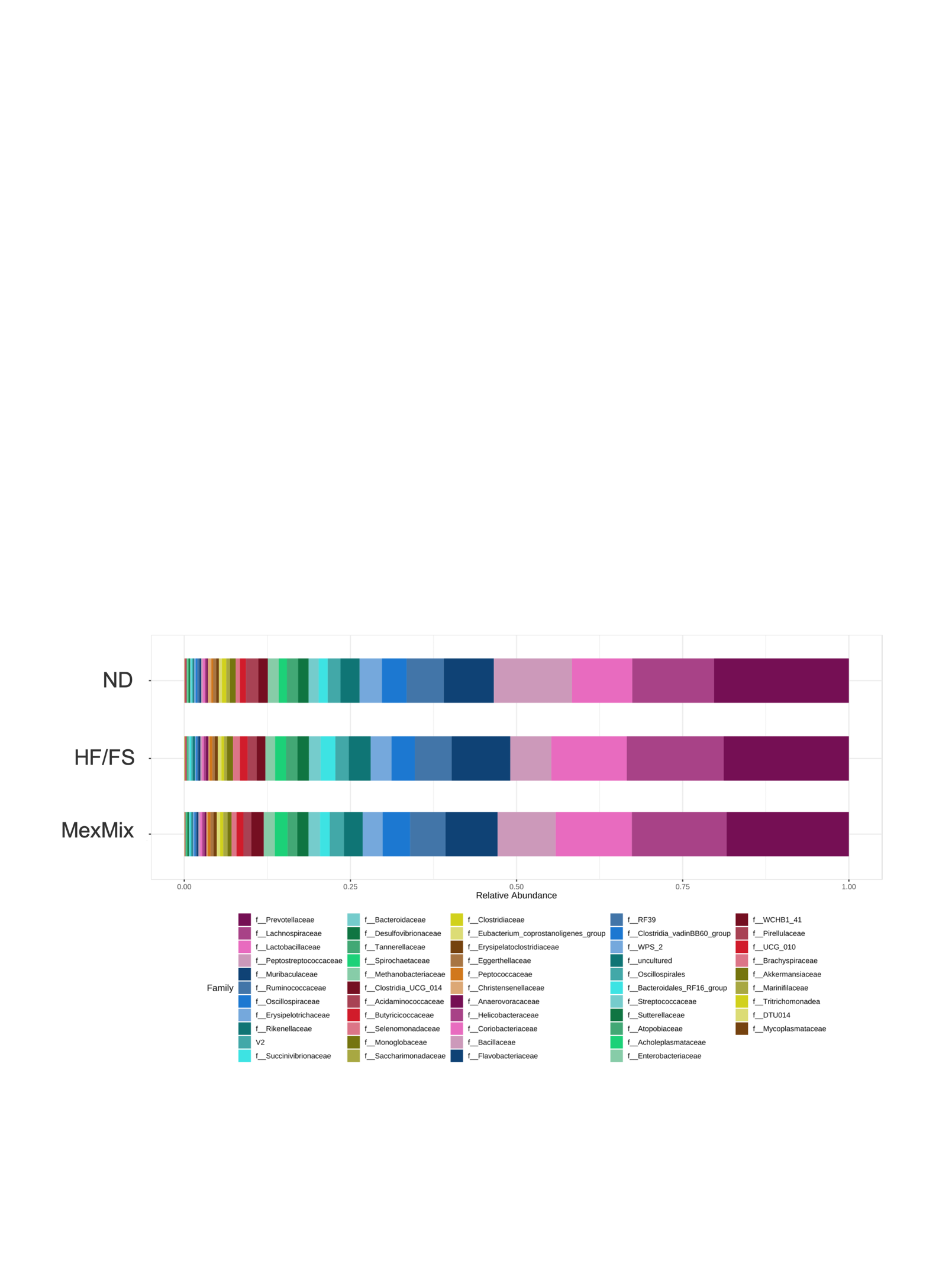
**

**Supplementary Figure 2:** Relative abundance of gut microbiota at family level.
